# Characterization of Mungbean *CONSTANS-LIKE* Genes and Functional Analysis of *CONSTANS-LIKE 2* in the Regulation of Flowering Time in *Arabidopsis*

**DOI:** 10.3389/fpls.2021.608603

**Published:** 2021-02-04

**Authors:** Chenyang Liu, Qianqian Zhang, Hong Zhu, Chunmei Cai, Shuai Li

**Affiliations:** ^1^Key Laboratory of Plant Biotechnology in Universities of Shandong Province, College of Life Sciences, Qingdao Agricultural University, Qingdao, China; ^2^College of Agronomy, Qingdao Agricultural University, Qingdao, China

**Keywords:** mungbean, flowering time, CONSTANS, *VrCOL*2, genome wide

## Abstract

*CONSTANS-LIKE* (*COL*) genes play important roles in the regulation of plant growth and development, and they have been analyzed in many plant species. However, few studies have examined *COL* genes in mungbean (*Vigna radiata*). In this study, we identified and characterized 31 mungbean genes whose proteins contained B-Box domains. Fourteen were designated as *VrCOL* genes and were distributed on 7 of the 11 mungbean chromosomes. Based on their phylogenetic relationships, *VrCOLs* were clustered into three groups (I, II, and III), which contained 4, 6, and 4 members, respectively. The gene structures and conserved motifs of the *VrCOL* genes were analyzed, and two duplicated gene pairs, *VrCOL1/VrCOL2* and *VrCOL8/VrCOL9*, were identified. A total of 82 *cis*-acting elements were found in the *VrCOL* promoter regions, and the numbers and types of *cis*-acting elements in each *VrCOL* promoter region differed. As a result, the expression patterns of *VrCOLs* varied in different tissues and throughout the day under long-day and short-day conditions. Among these *VrCOL* genes, *VrCOL2* showed a close phylogenetic relationship with *Arabidopsis thaliana CO* and displayed daily oscillations in expression under short-day conditions but not long-day conditions. In addition, overexpression of *VrCOL2* accelerated flowering in *Arabidopsis* under short-day conditions by affecting the expression of the flowering time genes *AtFT* and *AtTSF*. Our study lays the foundation for further investigation of *VrCOL* gene functions.

## Introduction

Flowering time is a key factor that influences crop growth and development, and crops achieve higher yields when they flower at the correct time. To regulate flowering time, crops sense the interactions between endogenous and environmental factors to determine the transition from vegetative to reproductive growth (Wickland and Hanzawa, 2015; Beinecke et al., 2018; Eom et al., 2018; Xu and Chong, 2018). Several functional pathways have been identified that regulate the switch from vegetative to reproductive development. These include the photoperiodic, vernalization, ambient temperature, plant hormone, and autonomous flowering pathways (Boss et al., 2004; Jack, 2004; Baurle and Dean, 2006; Wickland and Hanzawa, 2015; Xu and Chong, 2018; Ronald and Davis, 2019; Taylor et al., 2019; Zhang et al., 2019). A number of genes in these pathways are known to be involved in flowering time regulation, including *CONSTANS-LIKE* (*COL*) genes, phosphatidyl ethanolamine-binding protein (PEBP) genes, and several members of the MADS-box gene family (Gangappa and Botto, 2014; Wickland and Hanzawa, 2015; Beinecke et al., 2018; del-Olmo et al., 2019; Jin et al., 2019, 2020; Jing et al., 2019; Lee et al., 2019; Nam et al., 2019; Ning et al., 2019; Parenicova et al., 2019).

*COL* genes belong to the zinc-finger transcription factor family and play central roles in plant growth and development (Khanna et al., 2009; Gangappa and Botto, 2014). COL proteins are identified based on their conserved structure, which includes one or two BBX (B-Box) domains and one CCT (CONSTANS, CO-like, and TIMING of CAB1) domain (Khanna et al., 2009; Gangappa and Botto, 2014). The BBX domain can be further divided into two types, B-Box1 and B-Box2, which are recognized by their consensus sequences and the distances between their zinc-binding residues, which are considered to be involved in protein-protein interactions (Khanna et al., 2009). The CCT domain has important functions in transcriptional regulation and nuclear protein transport (Robson et al., 2001; Khanna et al., 2009; Yan et al., 2011; Gangappa and Botto, 2014). The COL proteins are grouped into three classes based on the number and type of their conserved domains. Classes I and II have two distinct BBX domains and one CCT domain, whereas class III has only one BBX and one CCT domain. Classes I, II, and III contain 6, 7, and 4 members in *Arabidopsis*, respectively. In addition, several COL proteins contain valine-proline (VP) motifs in their C termini (Khanna et al., 2009; Gangappa and Botto, 2014).

Among these *COL* members, *AtCO* (*AtBBX1*) and its homologs are well studied in many plant species (Khanna et al., 2009; Gangappa and Botto, 2014; Luo et al., 2018; Luccioni et al., 2019; Serrano-Bueno et al., 2020). *AtCO* is expressed in a rhythmic manner and coordinates light pathway and circadian clock signal inputs in *Arabidopsis* (Putterill et al., 1993, 1995; Andres and Coupland, 2012; Song et al., 2013). Thus, *AtCO* plays an important role in the regulation of flowering time by the photoperiod-dependent pathway. *Atco* mutants exhibit delayed flowering time under long-day conditions (LD), but under short-day conditions (SD), their flowering times are similar to those of wild-type plants. By contrast, *AtCO* overexpression plants show early flowering time under both LD and SD conditions (Khanna et al., 2009; Gangappa and Botto, 2014). The AtCO protein binds to *cis*-acting elements in the promoter region of the flowering activator *FLOWERING LOCUS T* (*AtFT*) to active *AtFT* expression. Moreover, AtCO is regulated by many flowering factors, such as AtGI (GIGANTEA), AtCDF1 (CYCLING DOF FACTOR 1) and AtFKF1 (FLAVIN BINDING, KELCH REPEAT, F-BOX1) (Imaizumi et al., 2005; Sawa et al., 2007). *OsHd1* (*Heading date 1*), the *AtCO* ortholog in rice, accelerates flowering under SD conditions but delays flowering under LD conditions through the regulation of the *AtFT* orthologs *OsHd3a* (*Heading date 3a*) and *OsRFT1* (*RICE FLOWERING LOCUS T1*) (Yano et al., 2000; Komiya et al., 2008, 2009). The soybean *AtCO* orthologs *GmCOL1*, *GmCOL2*, *GmCOL3*, and *GmCOL4* can complement the late flowering phenotype of *Atco* mutants (Wu et al., 2014). In addition to their functions in flowering time and circadian clock regulation, some COL proteins are also involved in abiotic or biotic stress responses, root development and stomatal opening (Khanna et al., 2009; Gangappa and Botto, 2014).

Mungbean is a diploid legume crop, and its seeds contain proteins and nutrients that are essential for human nutrition (Keatinge et al., 2011). The cultivated mungbean is thought to have been domesticated in India, from which it then spread to other areas (Fuller, 2007). Mungbean is considered to be an SD crop, and flowering time is a critical factor influencing its production (Vas Aggarwal and Poehlman, 1977; Imrie, 1996; Kim et al., 2015). Mungbean plants produce a large number of flowers, but only a few set pods. Approximately 70–90% of the flowers are shed, mainly the later-formed flowers of the racemes (Kumari and Verma, 1983; Mondal et al., 2011). Thus, it has been suggested that the prevention of late flowering is an important way to increase mungbean yield (Isobe et al., 1995; Kuroda et al., 1998; Mondal et al., 2011). The sequencing of the mungbean genome provides genetic resources for the investigation of gene functions (Kang et al., 2014), and the study of mungbean flowering time genes can therefore provide essential information for further modification of mungbean cultivars to increase yield. Until now, there has been limited information on the functions of genes involved in mungbean flowering time regulation. In this study, we identified mungbean *COL* genes and investigated their characteristics, including chromosomal distributions, gene structures, *cis*-acting elements and gene expression patterns. We also analyzed the functions of *VrCOL2* in the regulation of flowering time. Our findings will provide useful information for further characterization of mungbean *COL* gene functions.

## Materials and Methods

### Plant Materials and Growth Conditions

The mungbean reference genome variety VC1973A was provided by Suk-Ha Lee at Seoul National University, Seoul, South Korea (Kang et al., 2014) and used for all experiments in this study. Mungbean seeds were geminated in tap water for 1 day and then planted in soil-filled pots. Seedlings were grown in growth chambers with 16 h 25°C light/8 h 25°C dark cycles for LD conditions and 10 h 25°C light/14 h 25°C dark cycles for SD conditions. Leaves of 5-week-old mungbean plants were sampled every 4 h after lights-on and used to analyze the diurnal rhythm of gene expression. Multiple tissues were collected from field-grown mungbean plants sown at the end of May in Qingdao, China, including roots, nodule roots, shoot apices, stems, leaves, flowers, pods and seeds (Shi et al., 2021). Tissues were collected in the afternoon (ZT 10–12) in early July for gene expression analysis, and all samples were stored at −80°C before RNA extraction. *Arabidopsis* plants were grown in growth chambers with 16 h 23°C light/8 h 21°C dark cycles for LD conditions and 10 h 23°C light/14 h 21°C dark cycles for SD conditions. Leaves of 2-week-old *Arabidopsis* were collected every 4 h after lights-on for gene expression analysis.

### Identification of Mungbean *VrCOL* Members

The amino acid sequences of *Arabidopsis* BBXs were used as blast queries against the National Center for Biotechnology Information (NCBI) and mungbean genome databases^1^ to search for mungbean VrBBX proteins (Kang et al., 2014). The presence of conserved BBX and CCT domains in candidate genes was confirmed using the Pfam database and InterPro program with default parameters (Finn et al., 2017; El-Gebali et al., 2019).

### Phylogenetic Analysis

The amino acid sequences of CO and COL proteins from *Arabidopsis*, soybean, *Medicago*, mungbean, rice, and maize were aligned using ClustalW2 (Oliver et al., 2005), and the resulting alignment was used to construct a phylogenetic tree in MEGA 7.0 using the neighbor-joining method with default parameters (Kumar et al., 2016). In addition, VrBBX proteins were aligned separately in ClustalW2 and used to construct a phylogenetic tree in MEGA 7.0 with the neighbor-joining method.

### Chromosomal Distribution and Duplication Analyses

The physical positions of *VrCOL* genes were obtained from NCBI, and a chromosomal location map was constructed using MapInspect software (Mike Lischke, Berlin, Germany). Duplicated gene pairs were identified using OrthoMCL software as described by Fischer et al. (2011) and Jin et al. (2020). The duplicated gene pairs were defined as having greater than 60% amino acid sequence similarity and were visualized using Circos software (Krzywinski et al., 2009).

### Analyses of Exon-Intron Organization, Conserved Domains, Sequence Logos, Protein Motifs, and *Cis*-Acting Elements

The genomic and CDS sequences of mungbean *VrCOL* genes were obtained from NCBI and used as inputs to the Gene Structure Display Server (GSDS) to analyze their gene structures (Hu et al., 2015). The full-length amino acid sequences of *VrCOL* proteins were used to analyze the positions of the conserved BBX and CCT domains using the InterPro program (Finn et al., 2017). The sequence logos of the conserved BBX1, BBX2, and CCT domains were analyzed using the WebLogo platform (Crooks et al., 2004). The conserved motifs present in the *VrCOL* proteins were identified using MEME tools, with an optimum motif width of 11–50 amino acid residues (Bailey et al., 2009). The *cis*-acting elements in each *VrCOL* promoter, 2 kb upstream of the initiation codon, were predicted by PlantCARE (Lescot et al., 2002).

### Plasmid Construction and Plant Transformation

To investigate the functions of *VrCOL2*, a 35S: CDS-*VrCOL2* plasmid was constructed. The *VrCOL2* CDS was amplified from the cDNA of the sequenced mungbean variety VC1973A using primers with *Xho*I and *Xba*I digestion site sequences. The resulting PCR fragment was digested by the restriction endonucleases *Xho*I and *Xba*I to generate sticky ends. The pPTN1171 vector was digested with *Xho*I and *Xba*I to generate a linearized plasmid (Ping et al., 2014). Then the *VrCOL2* and pPTN1171 fragments were ligated using T4 DNA ligase (Promega). The constructed plasmid was verified by sequencing. It was then introduced into *Arabidopsis* using the floral dip method (Bent, 2006), and successful transformation was confirmed by PCR. All primers are listed in Supplementary Table 1.

### RNA Extraction and Transcription Analysis

RNA isolation and quantitative real-time PCR (qRT–PCR) analysis were carried out as described in Li et al. (2019). Gene expression levels were normalized to an *Actin* gene from mungbean (*Vradi03g00210*) (Li et al., 2019). Each sample was analyzed using three biological replicates. All primers are listed in Supplementary Table 1.

## Results

### Identification of *VrCOL* Genes in Mungbean

**FIGURE 1:**
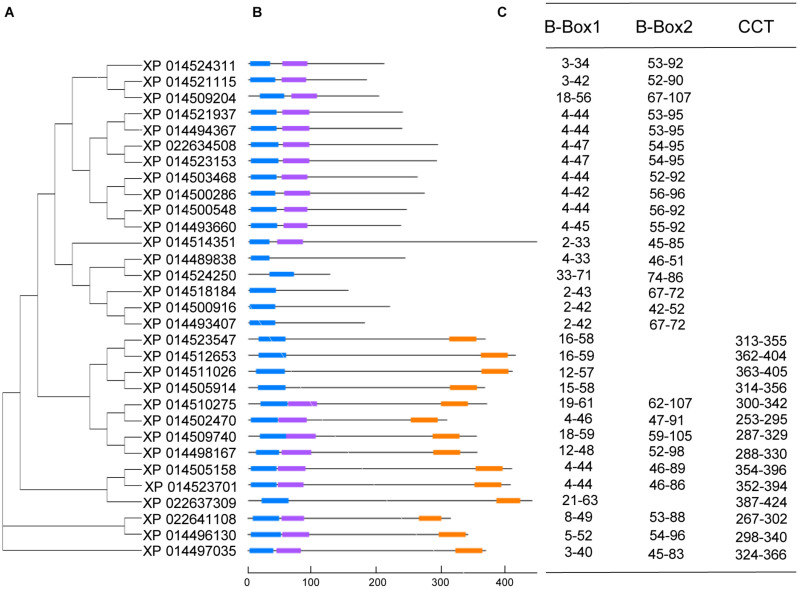
Phylogenetic relationships and conserved domain analyses of the VrBBX proteins. **(A)** Phylogenetic relationship analysis of 31 VrBBX proteins. The amino acid sequences of mungbean proteins containing BBX domains were used to construct a phylogenetic tree with the neighbor-joining method. **(B)** The positions of conserved BBX1, BBX2, and CCT domains in the VrBBX proteins. The blue, purple and orange boxes in each VrBBX protein indicate the BBX1, BBX2, and CCT domains, respectively. **(C)** The positions of the conserved BBX1, BBX2, and CCT domains in each VrBBX protein.

**FIGURE 2:**
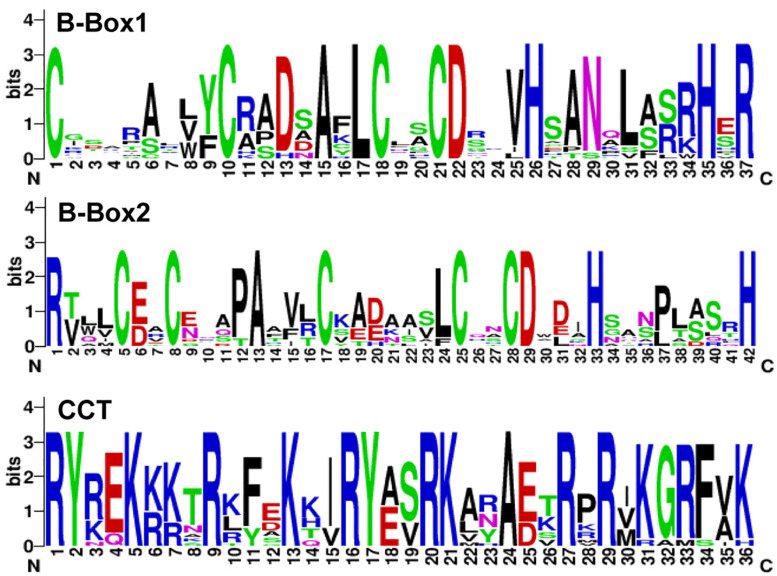
Sequence logos of the BBX1, BBX2, and CCT domains of *VrCOL* proteins. The conserved domains were analyzed using the WebLogo platform. The amino acid sequences of each conserved domain are presented on the *x*-axis, and the height of each letter corresponds to the conservation of each residue.

To search for mungbean *VrCOL* genes, we first identified mungbean proteins that contained BBX domains. The amino acid sequences of the conserved BBX domain (PF00643) and of *Arabidopsis* BBX proteins were used as blast queries against the mungbean genome database at NCBI. The presence of conserved BBX domains in each candidate mungbean gene was confirmed using Pfam and InterPro software, and a total of 31 *VrBBX* genes were identified in the mungbean genome (Figure 1). Among the VrBBX proteins, 17 contained only BBX domains, and 14 contained both BBX and CCT domains. The latter were designated *VrCOL* proteins (Figure 1 and Table 1). We then analyzed the numbers and types of BBX and CCT domains in the *VrCOL* proteins, and found two distinct BBX domains (BBX1 and BBX2) and one CCT domain (Supplementary Figure 1). Sequence logos of the BBX1 (CX_2_CX_8_CX_4_AXLCX_2_CDX_3_HX_8_HXR), BBX2 (CX_2_CX_4_AX_3_CX_7_CX_2_CDX_3_HX_8_H), and CCT (RYX_2_ KX_3_RX_3_KX_2_RYX_2_RKX_2_AX_2_RXR) domains were produced using WebLogo (Figure 2 and Supplementary Figure 1). Nine *VrCOL* proteins contained one BBX1, one BBX2, and one CCT domain, and five *VrCOL* proteins contained one BBX1 and one CCT domain (Figure 1 and Table 1).

**TABLE 1:** *VrCOL* genes identified in mungbean genome.

Gene ID	Genomic length/bp	CDS/bp	No. of AA	pI	Mol.Wt/Da	GC%	Chr	Strand	Gene names
XP_014498167	2,481	1,074	357	5.82	39,253.5	42.93	4	−	*VrCOL1*
XP_014509740	2,195	1,071	356	5.27	39,685.26	40.68	1	−	*VrCOL2*
XP_014502470	1,506	933	310	7.01	33,756.82	50.13	5	−	*VrCOL3*
XP_014510275	1,778	1,119	372	6.11	40,396.31	50.39	7	−	*VrCOL4*
XP_014511026	3,495	1,239	412	4.95	46,524.78	36.89	1	+	*VrCOL5*
XP_014505914	2,175	1,110	369	5.64	41,395.12	40.37	7	+	*VrCOL6*
XP_014512653	2,060	1,254	417	5.28	46,964.34	43.83	8	−	*VrCOL7a*
XP_014523547	1,960	1,113	370	9.22	42,031.83	40.10	N/A	+	*VrCOL7b*
XP_014505158	8,864	1,236	411	5.21	45,045.34	34.64	6	+	*VrCOL8*
XP_014523701	14,007	1,230	409	4.86	44,487.59	40.33	5	+	*VrCOL9*
XP_022637309	4,494	1,329	442	6.47	48,806.9	42.28	5	+	*VrCOL10*
XP_014496130	4,536	1,035	344	6.47	38,345.14	45.16	3	−	*VrCOL11*
XP_022641108	3,819	954	317	6.82	35,966.39	40.02	8	−	*VrCOL12*
XP_014497035	3,750	1,119	372	7.00	41,848.9	39.46	4	+	*VrCOL13*

*Chr, chromosome number; AA, amino acid; Mol.Wt, molecular weight; pI, isoelectric point; N/A, not applicable.*

Multiple characteristics of the *VrCOL* members were analyzed based on their genomic and protein sequences (Table 1). The genomic lengths of *VrCOL* genes ranged from 1,506 (*XP_014502470*) to 14,007 bp (*XP_014523701*), the CDS lengths ranged from 933 (*XP_014502470*) to 1,329 bp (*XP_022637309*), and the amino acid numbers ranged from 310 to 442. The isoelectric points of *VrCOL* proteins varied from 4.86 (XP_014523701) to 9.22 (XP_014523547), and their molecular weights ranged from 33,756.82 Da (XP_014502470) to 48,806.9 Da (XP_022637309). The GC content, which influences gene stability to some degree, ranged from 34.64 to 50.39%, and 12 of the 14 *VrCOL* genes had lower than 50% GC content (Table 1).

### Phylogenetic Analysis of the *VrCOL* Proteins

**FIGURE 3:**
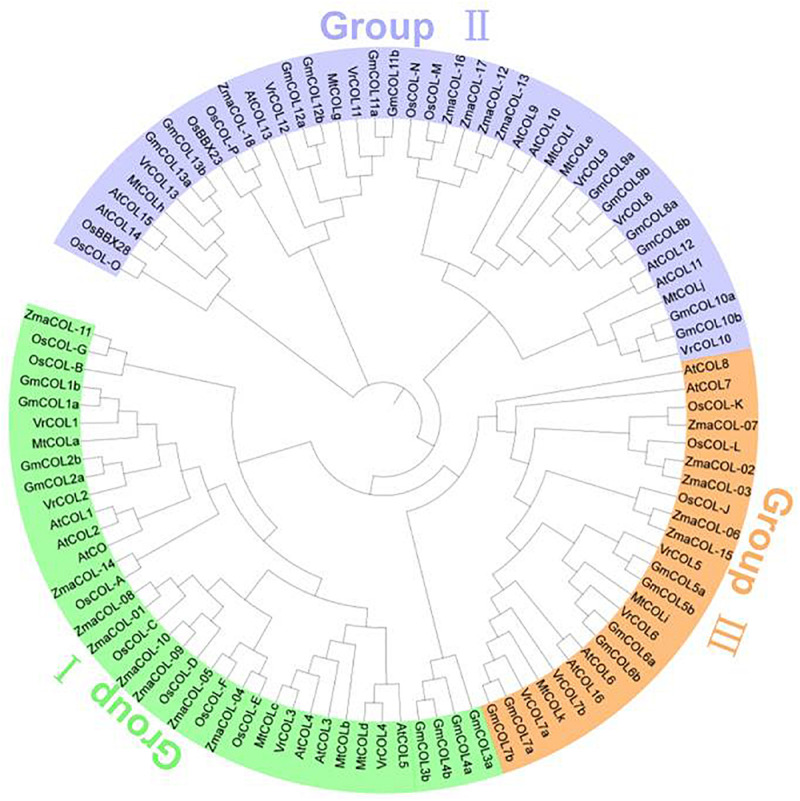
Evolutionary relationships among *VrCOL* proteins and COL proteins from other species. The amino acid sequences of COL proteins from *Arabidopsis*, soybean, *Medicago*, mungbean, rice and maize were used to construct a phylogenetic tree in MEGA 7.0 with the neighbor-joining method. *VrCOL* proteins are grouped into three classes and indicated with different colors.

To analyze the evolutionary relationships among the *VrCOL* genes and obtain information from well-studied *CO* homologs in other species, a phylogenetic tree was constructed using 17 *Arabidopsis*, 26 soybean, 11 *Medicago*, 16 rice, 18 maize, and 14 mungbean CO and COL proteins (Gangappa and Botto, 2014; Wu et al., 2014, 2017; Hu et al., 2018). The *VrCOL* genes were named *VrCOL1* to *VrCOL13* based on their phylogenetic relationships with their soybean orthologs (Figure 3 and Table 1). The COL proteins were grouped into three classes based on their phylogenetic relationships (Khanna et al., 2009; Gangappa and Botto, 2014; Figure 3). Classes I, II, and III contained 4, 6, and 4 *VrCOL* members, respectively (Figure 3). The BBX1 and BBX2 domains were located close to one another in the class I and II proteins, with the exception of *VrCOL*10 (Figure 1), whereas class III proteins contained only one BBX domain (Figures 1, 3). Among these *VrCOL* members, *VrCOL*1 and *VrCOL*2 showed close relationships to *Arabidopsis* AtCO, soybean GmCOL1a, GmCOL1b, GmCOL2a, and GmCOL2b and rice OsHd1 (OsCOL-A), all of which have documented roles in the regulation of flowering time (Khanna et al., 2009; Gangappa and Botto, 2014; Wu et al., 2014). This result suggests that *VrCOL*1 and *VrCOL*2 may play critical roles in the flowering time regulation of mungbean.

### Gene Structures and Conserved Motifs of the *VrCOL* Genes

**FIGURE 4:**
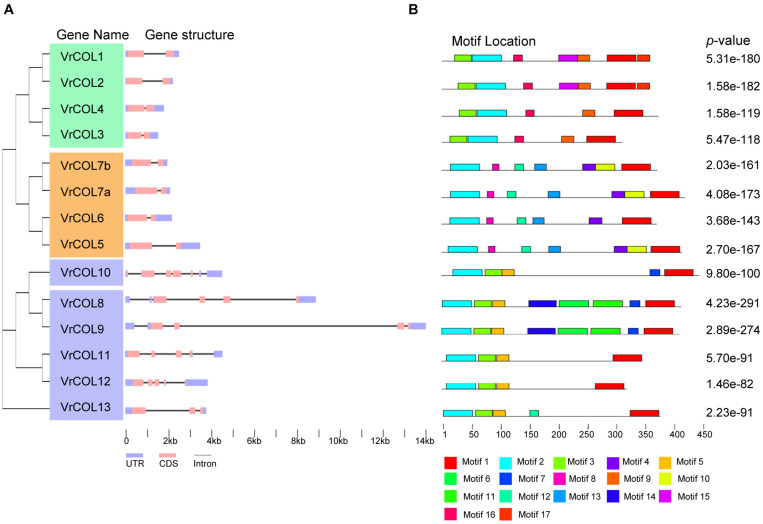
Gene structures and conserved motifs of the *VrCOL* proteins. **(A)** Exon-intron organization of the *VrCOL* genes. The length of each *VrCOL* gene is indicated, and the blue boxes, pink boxes and black lines indicate UTRs, exons and introns, respectively. **(B)** Conserved motifs of the *VrCOL* proteins. Conserved motifs were analyzed using MEME tools, and different motifs are indicated by different colored boxes.

To investigate the gene structures of the *VrCOL* genes, we downloaded their genomic and CDS sequences from NCBI and analyzed them using the GSDS program (Hu et al., 2015). All the *VrCOL* members contained 5′ UTR and 3′ UTR regions. Their exon numbers ranged from two to six, and their intron numbers ranged from one to six. All the group I and III *VrCOL* members contained two exons and one intron (Figure 4). By contrast, group II members contained various numbers of exons (3–6) and introns (2–6), suggesting potential functional diversity among these genes (Figure 4). To further investigate the conservation and diversity of *VrCOL* protein structures, we analyzed putative protein motifs in the *VrCOL*s. A total of 17 distinct motifs were identified, and all *VrCOL* proteins contained motifs 1 and 2, which appeared to represent the conserved BBX1 and CCT domains, respectively (Figure 4 and Supplementary Figure 2). Most members of the same class shared some conserved motifs. For example, class I proteins shared motifs 1, 2, 3, 9, and 16, class II members shared motifs 1, 2, 3, and 5, and class III members shared motifs 1, 2, 4, 8, 12, and 13 (Figure 4).

### Chromosomal Distribution and Duplication Analysis of the *VrCOL* Genes

**FIGURE 5:**
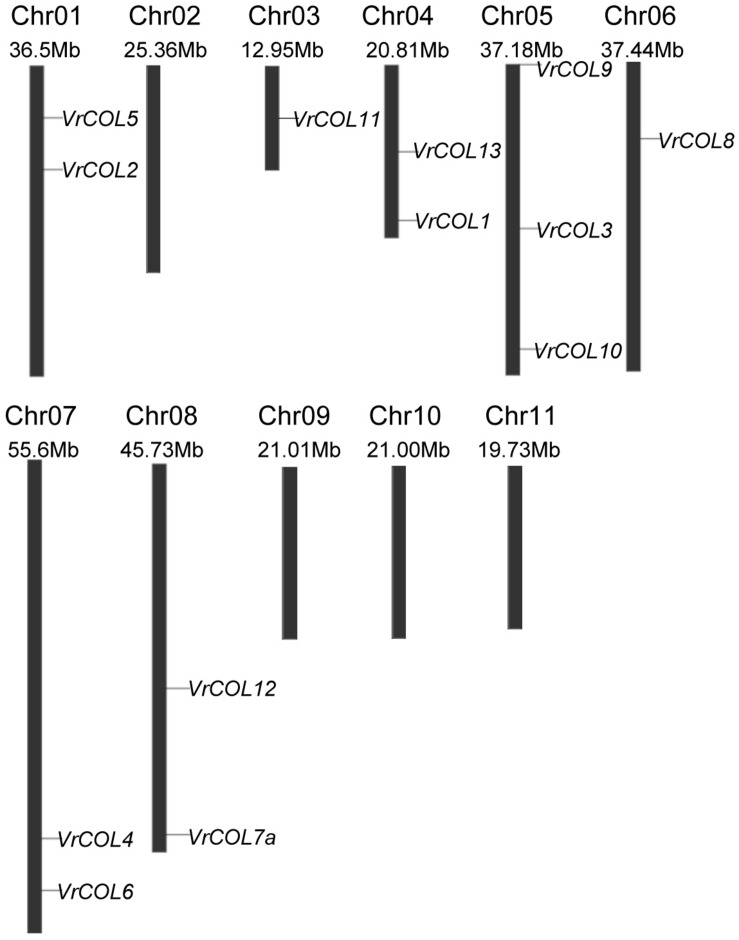
Chromosomal locations of the *VrCOL* genes. Chromosome number, chromosome length, and the positions of *VrCOL*s on the chromosome are indicated.

Some genes have evolved from common ancestors, and the chromosomal locations of *COL* genes may provide insight into changes in gene distribution during evolution. To visualize the chromosomal locations of the *VrCOL* genes, we mapped them to their physical positions in the mungbean genome. *VrCOL7b* was discarded due to a lack of related positional information. Seven of the 14 *VrCOL* genes were located on the positive strand. Seven of the 11 mungbean chromosomes contained *VrCOL* genes, with the exception of chromosomes 2, 9, 10, and 11 (Figure 5 and Table 1). Chromosome 5 contained the greatest number of *VrCOL* genes (three), followed by chromosomes 1, 4, 7, and 8, with two genes on each. In addition, most of the *VrCOL* genes were located on the relatively long chromosomes (1, 5, 6, 7, and 8). Only three members (*VrCOL1*, *VrCOL11*, and *VrCOL13*) were located on the relatively short chromosomes 3 and 4 (Figure 5).

**FIGURE 6:**
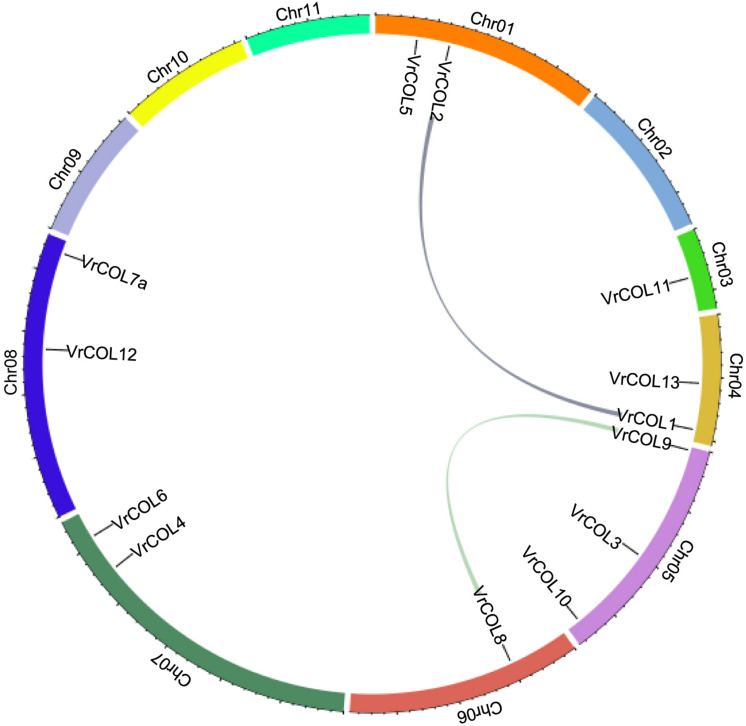
Duplication analyses of *VrCOL* proteins. The positions of *VrCOL* genes on the chromosomes are presented, and duplicated gene pairs are connected by colored lines.

Mungbean has experienced one round of whole-genome duplication that produced many duplicated gene pairs (Kang et al., 2014; Li et al., 2019). To investigate the evolutionary relationships among the *VrCOLs*, we searched for duplicated gene pairs among them. Two interchromosomal duplication events were identified in chromosomes 1, 4, 5, and 6, producing the duplicated gene pairs *VrCOL1/VrCOL2* and *VrCOL8/VrCOL9* (Figure 6). The duplicated genes were clustered together in the phylogenetic tree (Figure 1). All the duplicated genes contained one BBX1, one BBX2 and one CCT domain and belonged to groups I and II; no duplicated gene pairs were found in group III. The duplicated genes *VrCOL1* and *VrCOL2* showed similar exon-intron organization and similar motifs, as did *VrCOL8* and *VrCOL9* (Figure 4), indicating that the duplicates may share similar functions.

### *Cis*-Acting Element Analysis of the *VrCOL* Promoter Regions

To predict the potential expression responses of *VrCOL* genes, we investigated the *cis*-acting elements in their promoters using PlantCARE (Lescot et al., 2002). A total of 82 *cis*-acting elements were found across the 14 *VrCOL* promoter regions (2 kb upstream of the initiation codon) (Supplementary Table 2). Forty-five of them had predicted functions, including six development-related elements, four environmental-stress-related elements, three site-binding-related elements, nine hormone-responsive elements, three promoter-related elements and twenty light-responsive elements (Table 2 and Supplementary Table 2). The various *VrCOL* promoter regions had different numbers and types of *cis*-acting elements, highlighting the functional diversity of these genes. All *VrCOL* promoters contained hormone-responsive elements, light-responsive elements and promoter-related elements. Light-responsive elements were the most abundant element in each *VrCOL* promoter, with the exception of *VrCOL8* (Table 2), indicating that *VrCOL* genes may play critical roles in light-dependent signaling pathways. Environmental-stress-related elements were the most abundant element in the *VrCOL8* promoter (nine elements), indicating that *VrCOL8* may function in stress response (Table 2). All the *VrCOL* genes contained the promoter-related elements CAAT-Box and TATA-Box, which are basic promoter components. Thirteen of the 14 *VrCOL*s contained the hormone-responsive elements CGTCA-motif and TGACG-motif and the light-responsive element Box 4 (Supplementary Table 2), suggesting potential functions of these genes in related signaling pathways.

**TABLE 2:** Numbers and types of *cis*-acting elements in each *VrCOL* promoter region.

Gene name	Development related elements	Environmental stress related elements	Hormone-responsive elements	Light-responsive elements	Promoter related elements	Site-binding related elements	Others
*VrCOL1*	0	3	4	11	2	0	18
*VrCOL2*	1	3	4	6	2	1	18
*VrCOL3*	2	1	4	8	2	0	19
*VrCOL4*	1	0	4	11	2	2	17
*VrCOL5*	1	3	3	6	2	0	17
*VrCOL6*	1	0	4	11	2	2	17
*VrCOL7a*	1	1	5	8	2	2	17
*VrCOL7b*	0	0	4	7	2	0	14
*VrCOL8*	1	9	4	8	2	0	13
*VrCOL9*	0	1	5	7	2	0	14
*VrCOL10*	1	2	4	6	2	1	14
*VrCOL11*	0	2	5	6	3	0	20
*VrCOL12*	0	3	4	7	2	1	16
*VrCOL13*	4	1	4	6	2	2	15

### Transcription Patterns of *VrCOL* Genes in Different Tissues

**FIGURE 7:**
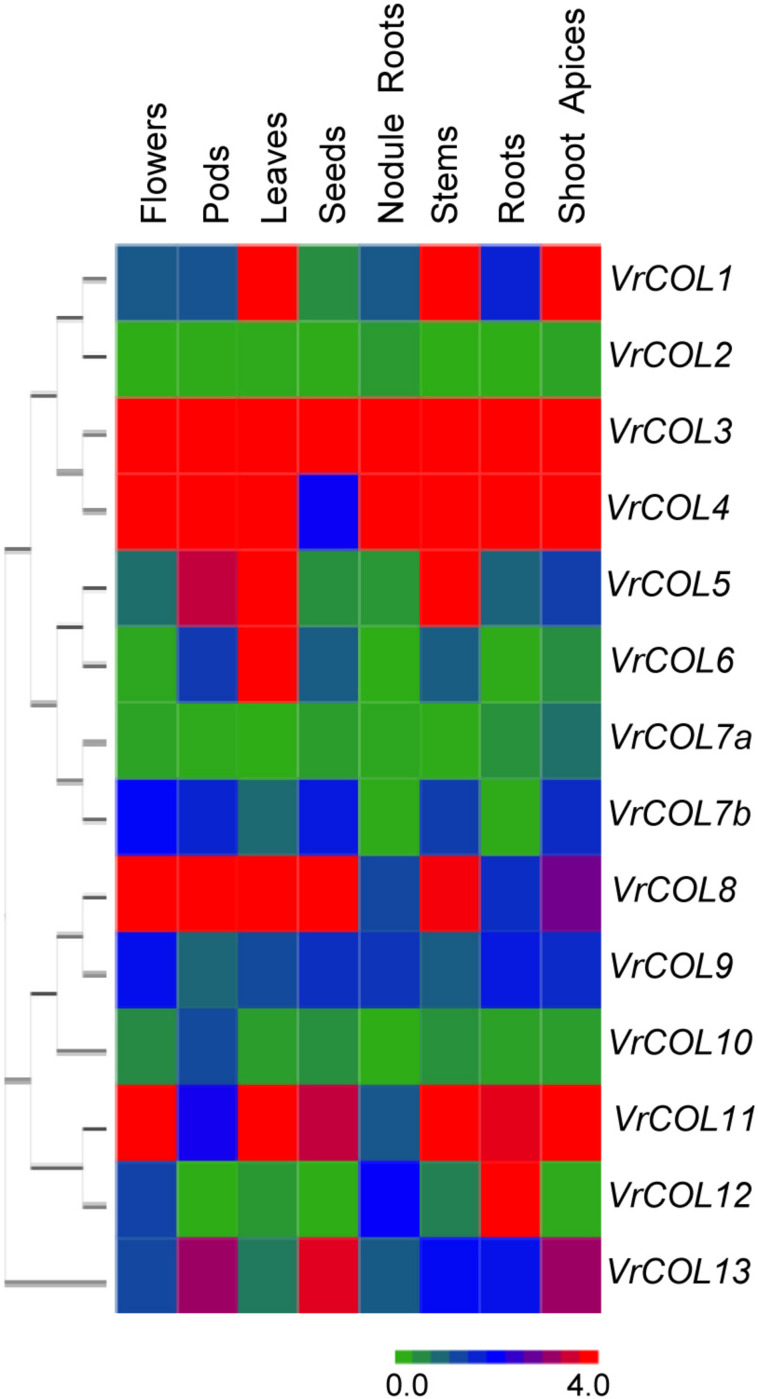
Relative expression levels of *VrCOL* genes in different tissues. The expression levels of *VrCOL* genes were analyzed by qRT–PCR. The expression level of *VrCOL1* in flowers was set to 1, and other values were adjusted accordingly. The gene expression results were visualized using a heatmap generated with Multiple Experiment Viewer 4.9.0 (Saeed et al., 2003). Different colors in the heatmap indicate different expression levels.

To shed light on the potential functions of *VrCOL* genes during plant development, we analyzed the expression of *VrCOL* genes in different tissues, including roots, nodule roots, shoot apices, stems, leaves, flowers, pods and seeds. *VrCOL* genes showed distinct expression patterns in different tissues (Figure 7). For example, *VrCOL3* was highly expressed in all tissues examined, whereas *VrCOL2* and *VrCOL7a* showed low expression in most tissues. Some genes were expressed at high levels in specific tissues, suggesting that they may have critical functions in those tissues. For example, *VrCOL6* showed high expression in leaves but low expression in nodule roots and flowers.

Duplicated genes may retain some common functions and evolve some new functions (Kondrashov et al., 2002; Wang et al., 2015). To investigate the conservation and diversity of duplicated genes, we also analyzed their tissue-specific expression patterns. *VrCOL1* and *VrCOL2* differed in their expression levels across all tissues examined, indicating that they may have different responses to the environment in these tissues. *VrCOL8* and *VrCOL9* showed similar expression levels in roots and nodule roots but different expression levels in other tissues (Figure 7 and Supplementary Figure 3).

### Diurnal Rhythm of *VrCOL* Gene Expression

**FIGURE 8:**
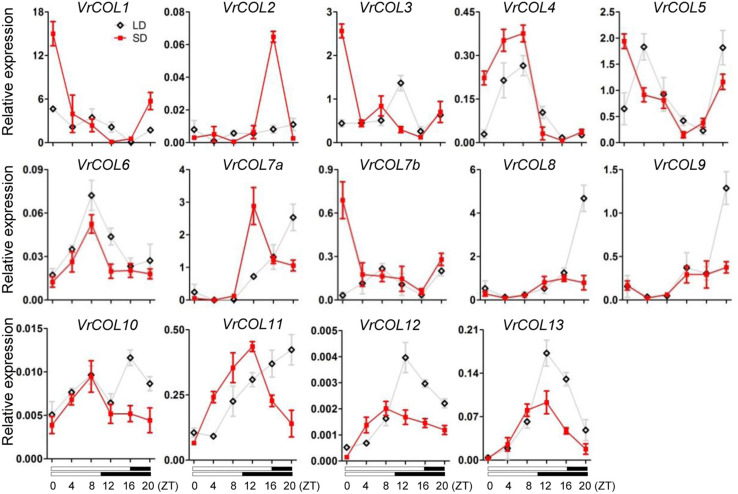
Relative expressions of *VrCOLs* in mungbean leaves throughout the day under SD and LD conditions. The SD condition was set as 8:00 am–6:00 pm light, 6:00 pm–8:00 am dark; the LD condition was set as 8:00 am–0:00 am light, 0:00 am–8:00 am dark. ZT, Zeitgeber Time. Expression level of *VrCOLs* was normalized to an *Actin* gene from mungbean. The gray and red lines indicate *VrCOL* expression levels under LD and SD conditions, respectively. Each sample was analyzed using three biological replicates.

In *Arabidopsis*, the expression levels of *CO*, *COL1*, and *COL2* are regulated by the circadian clock and show diurnal oscillations (Suárez-López et al., 2001; Gangappa and Botto, 2014). We therefore investigated whether *VrCOL* genes exhibited diurnal expression rhythms in mungbean leaves under LD and SD conditions. Gene expression analysis revealed that *VrCOL4*, *VrCOL6*, *VrCOL12*, and *VrCOL13* showed daily oscillations under both LD and SD conditions, whereas *VrCOL1*, *VrCOL2*, *VrCOL5*, *VrCOL7a*, *VrCOL7b*, *VrCOL10*, and *VrCOL11* showed daily oscillations only under SD conditions (Figure 8). The duplicated genes *VrCOL8* and *VrCOL9* exhibited similar expression patterns under both LD and SD conditions, whereas *VrCOL1* and *VrCOL2* showed distinct expression patterns under both LD and SD throughout the day (Figure 8).

### Overexpression of *VrCOL2* Accelerates Flowering in Arabidopsis Under SD Conditions

**FIGURE 9:**
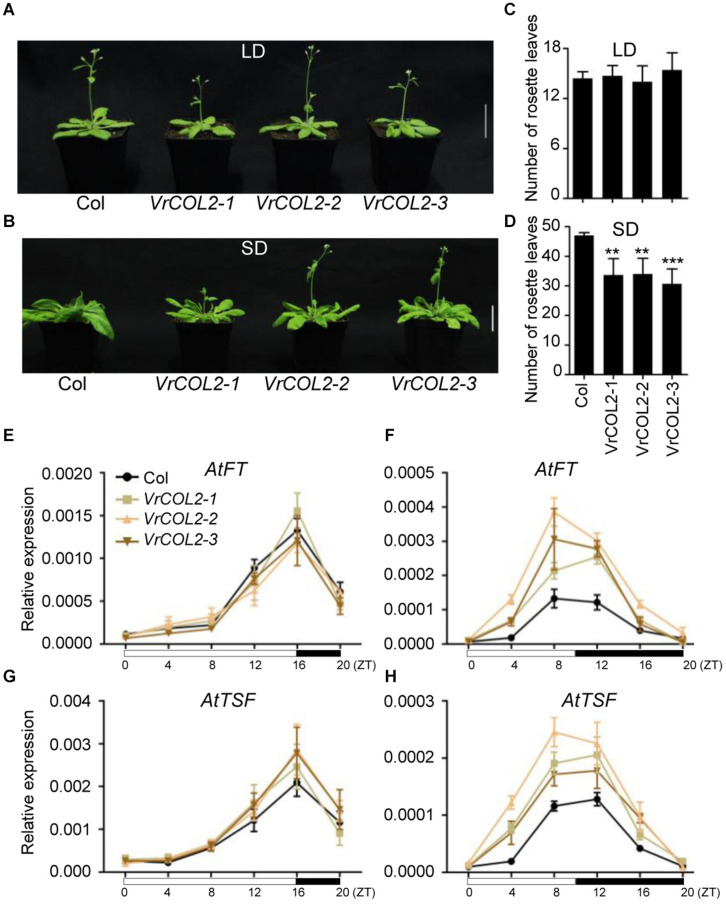
Overexpression of *VrCOL2* accelerates flowering under SD conditions in *Arabidopsis*. Flowering time phenotypes of three *VrCOL2* overexpression transgenic lines and wild-type *Arabidopsis* (Col) plants grown under LD **(A)** and SD conditions **(B)**. Bar = 4 cm. The rosette leaf numbers of *VrCOL2* overexpression transgenic lines and wild-type plants grown under LD **(C)** and SD conditions **(D)** as shown in panels **(A,B)**. The expression levels of *AtFT* and *AtTSF* throughout the day in *VrCOL2* transgenic lines and wild-type plants under LD **(E,G)** and SD **(F,H)** conditions. Leaves of 2-week-old *VrCOL2* overexpression transgenic lines and wild-type plants were sampled every 4 h after lights-on. Expression levels of *AtFT* and *AtTSF* were normalized to an *Actin* gene from *Arabidopsis*. The SD condition was set as 8:00 am–6:00 pm light, 6:00 pm–8:00 am dark; the LD condition was set as 8:00 am–0:00 am light, 0:00 am–8:00 am dark.

*VrCOL1* and *VrCOL2* displayed close phylogenetic relationships with *AtCO* (Figure 1), and the amino acid sequences of *VrCOL*1 and *VrCOL*2 showed 49.35 and 50.93% similarities with AtCO, respectively. We speculated that *VrCOL1* and *VrCOL2* might influence flowering time in mungbean, and we therefore first analyzed the function of *VrCOL2* in the regulation of flowering time in *Arabidopsis* in this study. To investigate the potential functions of *VrCOL2* in flowering time regulation, *VrCOL2* was transformed into *Arabidopsis* under the control of the 35S promoter. The empty vector was also transformed into *Arabidopsis*, and the transgenic plants showed no differences from wild type under both LD and SD conditions (Supplementary Figure 4). The *VrCOL2* transgenic *Arabidopsis* lines showed high levels of *VrCOL2* expression (Supplementary Figure 5). The wild-type *Arabidopsis* plants and three *VrCOL2* overexpression lines exhibited approximately 14 rosette leaves after bolting under LD conditions, indicating that they had similar flowering times. By contrast, the wild-type *Arabidopsis* plants and three *VrCOL2* overexpression lines showed approximately 47, 34, 34, and 31 rosette leaves after bolting under SD conditions, suggesting that *VrCOL2* transgenic plants had earlier flowering times than wild-type plants (Figure 9). These results indicated that *VrCOL2* regulated flowering time through a photoperiod-dependent pathway.

*AtFT* and *AtTSF* accelerate flowering and are regulated by *AtCO* in *Arabidopsis* (Khanna et al., 2009; Gangappa and Botto, 2014), and we therefore investigated the expression of *AtFT* and *AtTSF* in wild-type and *VrCOL2* transgenic plants under LD and SD conditions throughout the day. *AtFT* and *AtTSF* showed similar expression levels in *VrCOL2* transgenic and wild-type plants in both light and dark conditions under LD treatment. By contrast, *AtFT* and *AtTSF* showed higher expression levels in *VrCOL2* transgenic plants at several time points than in wild-type plants under SD conditions (Figure 9). These results further support the conclusion that *VrCOL2* is involved in flowering time regulation under SD conditions.

## Discussion

In recent decades, the investigation of *CO* and *COL* genes in many plant species has greatly increased our knowledge about the molecular mechanisms of flowering time regulation, stress response and root development (Khanna et al., 2009; Gangappa and Botto, 2014). Mungbean is a globally important legume crop, and the mechanisms of its flowering time regulation are still largely unknown. In this study, we identified and characterized 14 *VrCOL* genes from the mungbean genome and investigated the function of *VrCOL2* in flowering time regulation.

The *Arabidopsis*, soybean, *Medicago*, and mungbean genomes contain 17, 26, 11, and 14 *CO* and *COL* members (Figure 1; Wong et al., 2014; Wu et al., 2014), and their genome sizes are 125 Mb (Initiative, 2000), 1100 Mb (Schmutz et al., 2010), 500 Mb (Young et al., 2011), and 579 Mb (Kang et al., 2014), respectively. Thus, genome size has no direct relationship with the number of *COL* genes in plants. Soybean has undergone two rounds of whole-genome duplication, whereas mungbean has experienced only one such duplication (Schmutz et al., 2010; Kang et al., 2014). As a result, the *COL* gene number in mungbean is approximately half that of soybean. Seven of the 11 (63.6%) mungbean chromosomes (Figure 5), seven of the eight (87.5%) *Medicago* chromosomes and 16 of the 20 (80.0%) soybean chromosomes contained *COL* genes (Wong et al., 2014; Wu et al., 2014), indicating that the distribution of *COL* genes has changed during evolution in legumes.

Plant genome evolution produces many duplicated gene pairs and provides resources for new gene functions (Kondrashov et al., 2002). Two duplicated gene pairs, *VrCOL1/VrCOL2* and *VrCOL8/VrCOL9* (Figure 6), were found among the mungbean *VrCOLs*. The duplicated genes showed close relationships in the phylogenetic tree and contained similar motifs (Figures 1, 4), indicating that they evolved from the same origin and may share similar functions. However, the duplicated gene pairs contained different numbers and types of *cis*-acting elements in their promoter regions and exhibited different expression levels in some tissues (Figure 7 and Table 2), suggesting that they might have evolved novel functions compared with their original gene. For example, *VrCOL8* and *VrCOL9* shared similar numbers of several *cis*-acting elements in their promoter regions, including promoter-related elements and site-binding related elements, but differed in the numbers of development-related elements, environmental-stress-related elements, hormone-responsive elements and light-responsive elements (Table 2 and Supplementary Table 2). *VrCOL8* and *VrCOL9* showed similar expression levels in roots and nodule roots, but their expression differed in flowers, pods, leaves, seeds, stems, and shoot apices (Figure 7 and Supplementary Figure 3). This result suggests that they may have retained some common functions from the original gene in roots and nodule roots but evolved novel functions in other tissues.

The expression of *VrCOL* genes in different tissues provides clues to their potential functions, and many *VrCOL* genes (such as *VrCOL6* and *VrCOL12*) showed tissue-specific expression patterns (Figure 7). However, several *VrCOL* genes (including *VrCOL2*, *VrCOL7a*, and *VrCOL10*) showed low expression levels in all tissues tested, despite the fact that their promoter regions contained many *cis*-acting elements (Figure 7, Table 2, and Supplementary Table 2). Gene expression is influenced by many factors. For example, many circadian clock and flowering time regulation genes are controlled by photoperiod. Their expression changes under different photoperiods and during the day and night (Suárez-López et al., 2001; Jack, 2004; Wickland and Hanzawa, 2015; Xu and Chong, 2018). For example, *VrCOL2* appeared to be a daily oscillation gene whose expression changed during the day under SD conditions but was low throughout the day under LD conditions (Figure 8). The different field-grown mungbean tissues were collected in the afternoon under relatively LD conditions in July, and that may explain why *VrCOL2* showed low expression levels in the tissue expression analysis (Figure 7).

*CO* and *CO*-homologous genes, such as *OsHd1*, play critical roles in flowering time regulation (Khanna et al., 2009; Gangappa and Botto, 2014). *VrCOL2* showed close relationships with *Arabidopsis CO*, soybean *GmCOL1a*, *GmCOL1b*, *GmCOL2a*, and *GmCOL2b* and rice *OsHd1* (*OsCOL-A*), and accelerated flowering under SD but not LD conditions in transgenic *Arabidopsis* lines (Figure 9). AtCO regulates *AtFT* and *AtTSF* to accelerate flowering (Putterill et al., 1993, 1995; Andres and Coupland, 2012; Song et al., 2013), and the expression of *AtFT* and *AtTSF* increased in *VrCOL2* transgenic *Arabidopsis* lines at several time points under SD but not LD conditions (Figure 9). Moreover, *VrCOL2* showed daily oscillations only under SD conditions, but not LD conditions (Figure 8), indicating that *VrCOL2* might only have functions under SD conditions. *VrCOL2* therefore affects the expression of downstream *AtFT* and *AtTSF* genes *via* photoperiod-dependent pathways. Moreover, AtCO protein accumulation is also regulated by the circadian clock. *AtCO* mRNA is highly abundant from late afternoon to dawn, but AtCO protein accumulates only in the late afternoon under LD conditions (Putterill et al., 1995; Shim and Imaizumi, 2015; Song et al., 2015; Shim et al., 2017). Although *VrCOL2* was controlled by the 35S promoter and expressed under both LD and SD conditions, the accumulation of *VrCOL2* proteins was unknown. Whether the accumulation of *VrCOL*2 protein depends on day length, in turn affecting flowering time by influencing *AtFT* and *AtTSF* expression requires further investigation. In addition, *AtCO* promotes flowering under LD conditions and suppresses flowering time under SD conditions (Luccioni et al., 2019), but rice *OsHd1* accelerates flowering under SD conditions and delays flowering under LD conditions (Yano et al., 2000; Komiya et al., 2008, 2009). Mungbean (Imrie, 1996; Kim et al., 2015) and rice are SD plants, and *Arabidopsis* is an LD plant, and this may explain why CO homologs have different functions in different plant species. These results suggest that *CO* and its homologs are involved in flowering time regulation under photoperiod-dependent pathways and have distinct roles in different plant species. Thus, in summer LD conditions, the expression of *VrCOL2* may be low and have little effect on the acceleration of flowering. In the autumn, as days become shorter, the expression of *VrCOL2* may increase and accelerate mungbean flowering. In addition, *VrCOL1* and *VrCOL2* form a duplicated gene pair and show a close relationship with one another (Figures 1, 6), and *VrCOL1* showed high expression levels in many tissues, indicating that *VrCOL1* may share similar functions to *VrCOL2* in flowering time regulation, a possibility that requires further investigation. Much more work is needed to fully elucidate the mechanisms by which *VrCOL2* affects flowering time and circadian clock regulation in mungbean.

## Data Availability Statement

The original contributions presented in the study are included in the article/Supplementary Material, further inquiries can be directed to the corresponding author/s.

## Author Contributions

SL conceived and designed the research. CL, QZ, HZ, and CC conducted the experiments and analyzed the data. SL and HZ wrote the manuscript. All authors read and approved the manuscript.

## Conflict of Interest

The authors declare that the research was conducted in the absence of any commercial or financial relationships that could be construed as a potential conflict of interest.

## Footnotes

1 http://plantgenomics.snu.ac.kr/mediawiki-1.21.3/index.php/Main_Page

## References

[B1] Andres F. Coupland G. (2012). The genetic basis of flowering responses to seasonal cues. *Nat. Rev Genet.* 13 627–639. 10.1038/nrg3291 22898651

[B2] Bailey T. Boden M. Buske F. Frith M. Grant C. Clementi L. (2009). MEME suite: tools for motif discovery and searching. *Nucleic Acids Res.* 37 W202–W208. 10.1093/nar/gkp335 19458158 PMC2703892

[B3] Baurle I. Dean C. (2006). The timing of developmental transitions in plants. *Cell* 125 655–664. 10.1016/j.cell.2006.05.005 16713560

[B4] Beinecke F. A. Grundmann L. Wiedmann D. R. Schmidt F. J. Caesar A. S. Zimmermann M. (2018). The FT/FD-dependent initiation of flowering under long-day conditions in the day-neutral species *Nicotiana tabacum* originates from the facultative short-day ancestor *Nicotiana tomentosiformis*. *Plant J.* 96 329–342. 10.1111/tpj.14033 30030859

[B5] Bent A. (2006). *Arabidopsis thaliana* floral dip transformation method. *Methods Mol. Biol.* 343 87–103. 10.1385/1-59745-130-4:87 16988336

[B6] Boss P. K. Bastow R. M. Mylne J. S. Dean C. (2004). Multiple pathways in the decision to flower: enabling, promoting, and resetting. *Plant Cell* 16(Suppl.) S18–S31. 10.1105/tpc.015958 15037730 PMC2643402

[B7] Crooks G. E. Hon G. Chandonia J. M. Brenner S. E. (2004). WebLogo: a sequence logo generator. *Genome Res.* 14 1188–1190. 10.1101/gr.849004 15173120 PMC419797

[B8] del-Olmo I. Poza-Viejo L. Piñeiro M. Jarillo J. A. Crevillén P. (2019). High ambient temperature leads to reduced *FT* expression and delayed flowering in *Brassica rapa* via a mechanism associated with H2AZ dynamics. *Plant J.* 100 343–356. 10.1111/tpj.14446 31257648

[B9] El-Gebali S. Mistry J. Bateman A. Eddy S. Luciani A. Potter S. (2019). The Pfam protein families database in 2019. *Nucleic Acids Res.* 47 427–432. 10.1093/nar/gky995 30357350 PMC6324024

[B10] Eom H. Park S. J. Kim M. K. Kim H. Kang H. Lee I. (2018). TAF15b, involved in the autonomous pathway for flowering, represses transcription of *FLOWERING LOCUS C*. *Plant J.* 93 79–91. 10.1111/tpj.13758 29086456

[B11] Finn R. Attwood T. Babbitt P. Bateman A. Bork P. Bridge A. (2017). InterPro in 2017–beyond protein family and domain annotations. *Nucleic Acids Res.* 45 190–199. 10.1093/nar/gkw1107 27899635 PMC5210578

[B12] Fischer S. Brunk B. P. Chen F. Gao X. Harb O. S. Iodice J. B. (2011). Using OrthoMCL to assign proteins to OrthoMCL-DB groups or to cluster proteomes into new ortholog groups. *Curr. Protoc. Bioinformatics* 35 1–19. 10.1002/0471250953.bi0612s35 21901743 PMC3196566

[B13] Fuller D. Q. (2007). Contrasting patterns in crop domestication and domestication rates: recent archaeobotanical insights from the old world. *Ann. Bot.* 100 903–924. 10.1093/aob/mcm048 17495986 PMC2759199

[B14] Gangappa S. N. Botto J. F. (2014). The *BBX* family of plant transcription factors. *Trends Plant Sci.* 19 460–470. 10.1016/j.tplants.2014.01.010 24582145

[B15] Hu B. Jin J. Guo A. Zhang H. Luo J. Gao G. (2015). GSDS 2.0: an upgraded gene feature visualization server. *Bioinformatics* 31 1296–1297. 25504850 10.1093/bioinformatics/btu817 PMC4393523

[B16] Hu T. Wei Q. Wang W. Hu H. Mao W. Zhu Q. (2018). Genome-wide identification and characterization of *CONSTANS-like* gene family in radish (*Raphanus sativus*). *PLoS One* 13:e0204137. 10.1371/journal.pone.0204137 30248137 PMC6152963

[B17] Imaizumi T. Schultz T. F. Harmon F. G. Ho L. A. Kay S. A. (2005). FKF1 F-Box protein mediates cyclic degradation of a repressor of *CONSTANS* in *Arabidopsis*. *Science* 309 293–297. 10.1126/science.1110586 16002617

[B18] Imrie B. C. (1996). “Mung bean,” in *The New Rural Industries: A Handbook for Farmers and Investors*, ed. Hyde K. (Canberra, ACT: Rural Industries Research and Development Corporation), 355–360.

[B19] Initiative A. G. (2000). Analysis of the genome sequence of the flowering plant *Arabidopsis thaliana*. *Nature* 408 796–815. 10.1038/35048692 11130711

[B20] Isobe K. Kokubun M. Tsuboki Y. (1995). Effects of soybean raceme-order on pod set and seed growth in three cultivars. *Jpn. J. Crop Sci.* 64 281–287. 10.1626/jcs.64.281

[B21] Jack T. (2004). Molecular and genetic mechanisms of floral control. *Plant Cell* 16(Suppl.) S1–S17. 10.1105/tpc.017038 15020744 PMC2643400

[B22] Jin H. Tang X. Xing M. Zhu H. Sui J. Cai C. (2019). Molecular and transcriptional characterization of phosphatidyl ethanolamine-binding proteins in wild peanuts *Arachis duranensis* and *Arachis ipaensis*. *BMC Plant Biol.* 19:484. 10.1186/s12870-019-2113-3 31706291 PMC6842551

[B23] Jin H. Xing M. Cai C. Li S. (2020). B-box proteins in *Arachis duranensis*: genome-wide characterization and expression profiles analysis. *Agronomy* 10:23 10.3390/agronomy10010023

[B24] Jing Y. Guo Q. Zha P. Lin R. (2019). The chromatin−remodeling factor PICKLE interacts with CONSTANS to promote flowering in *Arabidopsis*. *Plant Cell Environ.* 42 2495–2507. 10.1111/pce.13557 30965386

[B25] Kang Y. J. Kim S. K. Kim M. Y. Lestari P. Kim K. H. Ha B. K. (2014). Genome sequence of mungbean and insights into evolution within *Vigna* species. *Nat. Commun.* 5:5443. 10.1038/ncomms6443 25384727 PMC4241982

[B26] Keatinge J. D. H. Easdown W. J. Yang R. Y. Chadha M. L. Shanmugasundaram S. (2011). Overcoming chronic malnutrition in a future warming world: the key importance of mungbean and vegetable soybean. *Euphytica* 180 129–141. 10.1007/s10681-011-0401-6

[B27] Khanna R. Kronmiller B. Maszle D. R. Coupland G. Holm M. Mizuno T. (2009). The *Arabidopsis B-box* zinc finger family. *Plant Cell* 21 3416–3420. 10.1105/tpc.109.069088 19920209 PMC2798317

[B28] Kim S. K. Nair R. M. Lee J. Lee S. H. (2015). Genomic resources in mungbean for future breeding programs. *Front. Plant Sci.* 6:626. 10.3389/fpls.2015.00626 26322067 PMC4530597

[B29] Komiya R. Ikegami A. Tamaki S. Yokoi S. Shimamoto K. (2008). *Hd3a* and *RFT1* are essential for flowering in rice. *Development* 135 767–774. 10.1242/dev.008631 18223202

[B30] Komiya R. Yokoi S. Shimamoto K. (2009). A gene network for long-day flowering activates *RFT1* encoding a mobile flowering signal in rice. *Development* 136 3443–3450. 10.1242/dev.040170 19762423

[B31] Kondrashov F. A. Rogozin I. B. Wolf Y. I. Koonin E. V. (2002). Selection in the evolution of gene duplications. *Genome Biol.* 3:RESEARCH0008. 10.1186/gb-2002-3-2-research0008 11864370 PMC65685

[B32] Krzywinski M. Schein J. Birol I. Connors J. Gascoyne R. Horsman D. (2009). Circos: an information aesthetic for comparative genomics. *Genome Res.* 19 1639–1645. 10.1101/gr.092759.109 19541911 PMC2752132

[B33] Kumar S. Stecher G. Tamura K. (2016). MEGA7: molecular evolutionary genetics analysis version 7.0 for bigger datasets. *Mol. Biol. Evol.* 33 1870–1874. 10.1093/molbev/msw054 27004904 PMC8210823

[B34] Kumari P. Verma S. K. (1983). Genotypic differences in flower production, shedding and yield in mungbean. *J. Agric. Sci.* 99 219–223. 10.1016/0147-5975(83)90022-1

[B35] Kuroda T. Saitoh K. Mahmood T. Yanagawa K. (1998). Differences in flowering habit between determinate and indeterminate types of soybean. *Plant Prod. Sci.* 1 18–24. 10.1017/S0007485320000280

[B36] Lee C. Kim S. Jin S. Susila H. Youn G. Nasim Z. (2019). Genetic interactions reveal the antagonistic roles of *FT/TSF* and *TFL1* in the determination of inflorescence meristem identity in *Arabidopsis*. *Plant J.* 99 452–464. 10.1111/tpj.14335 30943325

[B37] Lescot M. Dehais P. Thijs G. Marchal K. Moreau Y. Van de Peer Y. (2002). PlantCARE, a database of plant *cis*-acting regulatory elements and a portal to tools for in silico analysis of promoter sequences. *Nucleic Acids Res.* 30 325–327. 10.1093/nar/30.1.325 11752327 PMC99092

[B38] Li S. Wang R. Jin H. Ding Y. Cai C. (2019). Molecular characterization and expression profile analysis of heat shock transcription factors in mungbean. *Front. Genet.* 9:736. 10.3389/fgene.2018.00736 30687395 PMC6336897

[B39] Liu C. Zhang Q. Zhu H. Cai C. Li S. (2020). Functional characterization of mungbean *CONSTANS-LIKE* genes reveals a key role for *CONSTANS-LIKE 2* in the control of flowering time in *A. thaliana* under short-day conditions. *Researchsquare* 10.21203/rs.3.rs-24842/v2

[B40] Luccioni L. Krzymuski M. Sánchez−Lamas M. Karayekov E. Cerdán P. D. Casal J. J. (2019). CONSTANS delays *Arabidopsis* flowering under short days. *Plant J.* 97 923–932. 10.1111/tpj.14171 30468542

[B41] Luo X. Gao Z. Wang Y. Chen Z. Zhang W. Huang J. (2018). The NUCLEAR FACTOR−CONSTANS complex antagonizes polycomb repression to de−repress *FLOWERING LOCUS T* expression in response to inductive long days in *Arabidopsis*. *Plant J.* 95 17–29. 10.1111/tpj.13926 29667247

[B42] Mondal M. M. A. Fakir M. S. A. Juraimi A. S. Hakim M. A. Islam M. M. Shamsuddoha A. T. M. (2011). Effects of flowering behavior and pod maturity synchrony on yield of mungbean [*Vigna radiata* (L.) Wilczek]. *Aust J. Crop Sci.* 5 945–953. 10.1111/j.1439-0523.2010.01842.x

[B43] Nam J. dePamphilis C. W. Ma H. Nei M. (2019). Antiquity and evolution of the MADS-box gene family controlling flower development in plants. *Mol. Biol. Evol.* 20 1435–1447. 10.1093/molbev/msg152 12777513

[B44] Ning Y. Chen Q. Lin R. Li Y. Li L. Chen S. (2019). The HDA19 histone deacetylase complex is involved in the regulation of flowering time in a photoperiod−dependent manner. *Plant J.* 98 448–464. 10.1111/tpj.14229 30828924

[B45] Oliver T. Schmidt B. Nathan D. Clemens R. Maskell D. (2005). Using reconfigurable hardware to accelerate multiple sequence alignment with clustalW. *Bioinformatics* 21 3431–3432. 10.1093/bioinformatics/bti508 15919726

[B46] Parenicova L. de Folter S. Kieffer M. Horner D. S. Favalli C. Busscher J. (2019). Molecular and phylogenetic analyses of the complete MADS-box transcription factor family in *Arabidopsis*: new openings to the MADS world. *Plant Cell* 15 1538–1551. 10.1105/tpc.011544 12837945 PMC165399

[B47] Ping J. Liu Y. Sun L. Zhao M. Li Y. She M. (2014). *Dt2* is a gain-of-function MADS-Domain factor gene that specifies semideterminacy in soybean. *Plant Cell* 26 2831–2842. 10.1105/tpc.114.126938 25005919 PMC4145117

[B48] Putterill J. Robson F. Lee K. Coupland G. (1993). Chromosome walking with YAC clones in *Arabidopsis*: isolation of 1700 kb of contiguous DNA on chromosome 5, including a 300 kb region containing the flowering-time gene *CO*. *Mol. Gen. Genet.* 239 145–157. 10.1007/BF00281613 8099710

[B49] Putterill J. Robson F. Lee K. Simon R. Coupland G. (1995). The *CONSTANS* gene of *Arabidopsis* promotes flowering and encodes a protein showing similarities to zinc finger transcription factors. *Cell* 80 847–857. 10.1016/0092-8674(95)90288-0 7697715

[B50] Robson F. Costa M. M. R. Hepworth S. R. Vizir I. Pineiro M. Reeves P. H. (2001). Functional importance of conserved domains in the flowering-time gene *CONSTANS* demonstrated by analysis of mutant alleles and transgenic plants. *Plant J.* 28 619–631. 10.1046/j.1365-313x.2001.01163.x 11851908

[B51] Ronald J. Davis S. J. (2019). Focusing on the nuclear and subnuclear dynamics of light and circadian signaling. *Plant Cell Environ.* 42 2871–2884. 10.1111/pce.13634 31369151

[B52] Saeed A. I. Sharov V. White J. Li J. Liang W. Bhagabati N. (2003). TM4: a free, open-source system for microarray data management and analysis. *Biotechniques* 34 374–378. 10.2144/03342mt01 12613259

[B53] Sawa M. Nusinow D. A. Kay S. A. Imaizumi T. (2007). FKF1 and GIGANTEA complex formation is required for day-length measurement in *Arabidopsis*. *Science* 318 261–265. 10.1126/science.1146994 17872410 PMC3709017

[B54] Schmutz J. Cannon S. Schlueter J. Ma J. Mitros T. Nelson W. (2010). Genome sequence of the palaeopolyploid soybean. *Nature* 463 178–183. 10.1038/nature08670 20075913

[B55] Serrano-Bueno G. Said F. E. de los Reyes P. Lucas−Reina E. I. Ortiz−Marchena M. I. Romero J. M. (2020). CONSTANS−FKBP12 interaction contributes to modulate photoperiodic flowering in *Arabidopsis*. *Plant J.* 101 1287–1302. 10.1111/tpj.14590 31661582

[B56] Shi R. Xu W. Liu T. Cai C. Li S. (2021). VrLELP controls flowering time under short-day conditions in *Arabidopsis*. *J. Plant Res.* 134 141–149. 10.1007/s10265-020-01235-7 33084994

[B57] Shim J. S. Imaizumi T. (2015). Circadian clock and photoperiodic response in *Arabidopsis*: from seasonal flowering to redox homeostasis. *Biochemistry* 54 157–170. 10.1021/bi500922q 25346271 PMC4303289

[B58] Shim J. S. Kubota A. Imaizumi T. (2017). Circadian clock and photoperiodic flowering in *Arabidopsis*: *CONSTANS* is a hub for signal integration. *Plant Physiol.* 173 5–15. 10.1104/pp.16.01327 27688622 PMC5210731

[B59] Song Y. H. Ito S. Imaizumi T. (2013). Flowering time regulation: photoperiod-and temperature-sensing in leaves. *Trends Plant Sci.* 18 575–583. 10.1016/j.tplants.2013.05.003 23790253 PMC3796012

[B60] Song Y. H. Shim J. S. Kinmonth-Schultz H. A. Imaizumi T. (2015). Photoperiodic flowering: time measurement mechanisms in leaves. *Annu. Rev. Plant Biol.* 66 441–464. 10.1146/annurev-arplant-043014-115555 25534513 PMC4414745

[B61] Suárez-López P. Wheatley K. Robson F. Onouchi H. Valverde F. Coupland G. (2001). CONSTANS mediates between the circadian clock and the control of flowering in *Arabidopsis*. *Nature* 410 1116–1120. 10.1038/35074138 11323677

[B62] Taylor C. M. Kamphuis L. G. Zhang W. Garg G. Berger J. D. Mousavi-Derazmahalleh M. (2019). INDEL variation in the regulatory region of the major flowering time gene *LanFTc1* is associated with vernalization response and flowering time in narrow−leafed lupin (*Lupinus angustifolius* L.). *Plant Cell Environ.* 42 174–187. 10.1111/pce.13320 29677403 PMC7379684

[B63] Vas Aggarwal D. Poehlman J. (1977). Effects of photoperiod and temperature on flowering in mungbean (*Vigna radiata* (L.) WILCZEK). *Euphytica* 26 207–219. 10.1007/BF00032086

[B64] Wang Z. Zhou Z. Liu Y. Liu T. Li Q. Ji Y. (2015). Functional evolution of phosphatidyl ethanolamine binding proteins in soybean and *Arabidopsis*. *Plant Cell* 27 323–336. 10.1105/tpc.114.135103 25663621 PMC4456927

[B65] Wickland D. P. Hanzawa Y. (2015). The *FLOWERING LOCUS T/TERMINAL FLOWER 1* gene family: functional evolution and molecular mechanisms. *Mol. Plant* 8 983–997. 10.1016/j.molp.2015.01.007 25598141

[B66] Wong A. C. S. Hecht V. F. G. Picard K. Diwadkar P. Laurie R. E. Wen J. (2014). Isolation and functional analysis of *CONSTANS-LIKE* genes suggests that a central role for *CONSTANS* in flowering time control is not evolutionarily conserved in *Medicago truncatula*. *Front. Plant Sci.* 5:486. 10.3389/fpls.2014.00486 25278955 PMC4166892

[B67] Wu F. Price B. W. Haider W. Seufferheld G. Nelson R. Hanzawa Y. (2014). Functional and evolutionary characterization of the *CONSTANS* gene family in short-day photoperiodic flowering in soybean. *PLoS One* 9:e85754. 10.1371/journal.pone.0085754 24465684 PMC3897488

[B68] Wu W. Zheng X. M. Chen D. Zhang Y. Ma W. Zhang H. (2017). *OsCOL16*, encoding a CONSTANS-like protein, represses flowering by up-regulating *Ghd7* expression in rice. *Plant Sci.* 260 60–69. 10.1016/j.plantsci.2017.04.004 28554475

[B69] Xu S. Chong K. (2018). Remembering winter through vernalisation. *Nat. Plants* 4 997–1009. 10.1038/s41477-018-0301-z 30478363

[B70] Yan H. Marquardt K. Indorf M. Jutt D. Kircher S. Neuhaus G. (2011). Nuclear localization and interaction with COP1 are required for STO/BBX24 function during photomorphogenesis. *Plant Physiol.* 156 1772–1782. 10.1104/pp.111.180208 21685177 PMC3149933

[B71] Yano M. Katayose Y. Ashikari M. Yamanouchi U. Monna L. Fuse T. (2000). Hd1, a major photoperiod sensitivity quantitative trait locus in rice, is closely related to the *Arabidopsis* flowering time gene *CONSTANS*. *Plant Cell* 12 2473–2483. 10.1105/tpc.12.12.2473 11148291 PMC102231

[B72] Young N. Debellé F. Oldroyd G. Geurts R. Cannon S. B. Udvardi M. K. (2011). The *Medicago* genome provides insight into the evolution of rhizobial symbioses. *Nature* 480 520–524. 10.1038/nature10625 22089132 PMC3272368

[B73] Zhang W. Yuan J. Cheng T. Tang M. J. Sun K. Song S. L. (2019). Flowering−mediated root−fungus symbiosis loss is related to jasmonate−dependent root soluble sugar deprivation. *Plant Cell Environ.* 42 3208–3226. 10.1111/pce.13636 31373013

