# Supplementary material for: Characterization of Mungbean CONSTANS-LIKE Genes and Functional Analysis of CONSTANS-LIKE 2 in the Regulation of Flowering Time in Arabidopsis
Source: Front Plant Sci. 2021 Feb 4;12:608603. doi: 10.3389/fpls.2021.608603 (PMC7890258; doi:10.3389/fpls.2021.608603)
Supplement: Supplementary file 2 [file Data_Sheet_1.docx]

Supplementary Material

Characterization of mungbean *CONSTANS-LIKE* genes and functional analysis of *CONSTANS-LIKE 2* in the regulation of flowering time in *Arabidopsis*

Chenyang Liu^1, #^, Qianqian Zhang^1, #^, Hong Zhu^2^, Chunmei Cai^1^, Shuai Li^1, *^

*Correspondence: Shuai Li: li2014shuai@qau.edu.cn


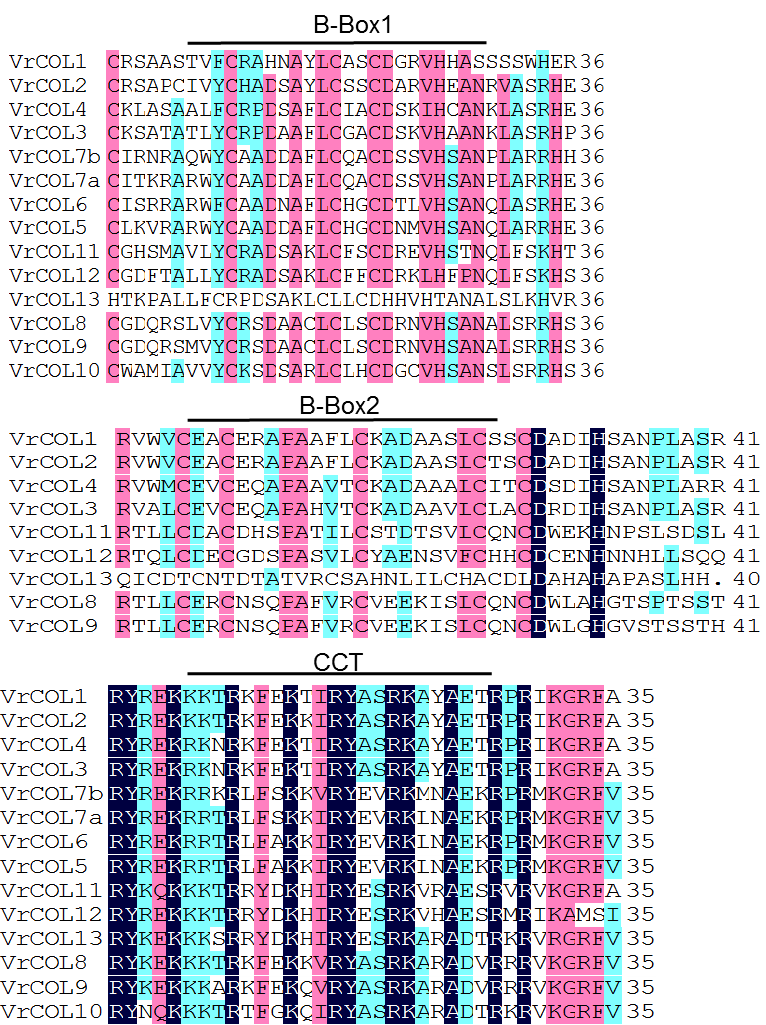


**Supplementary Figure S1 Sequence alignments of conserved B-Box1, B-Box2 and CCT domains of VrCOL proteins.**


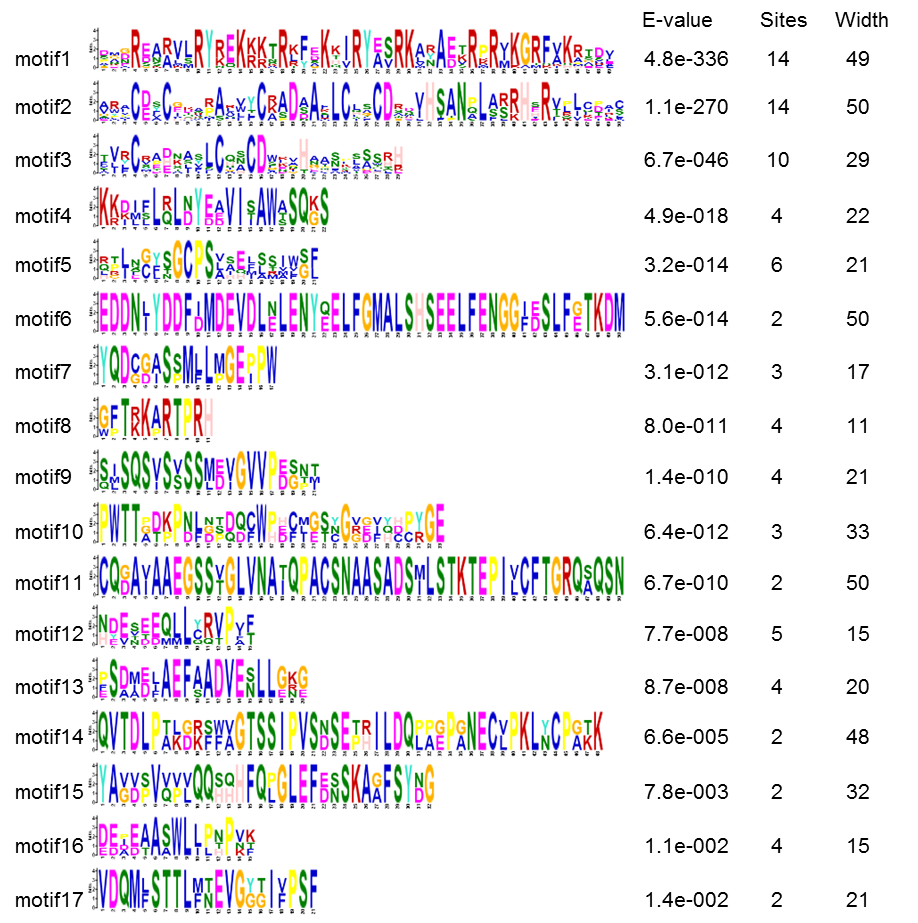


**Supplementary Figure S2 Sequence logos of 17 distinct motifs in VrCOL proteins.**


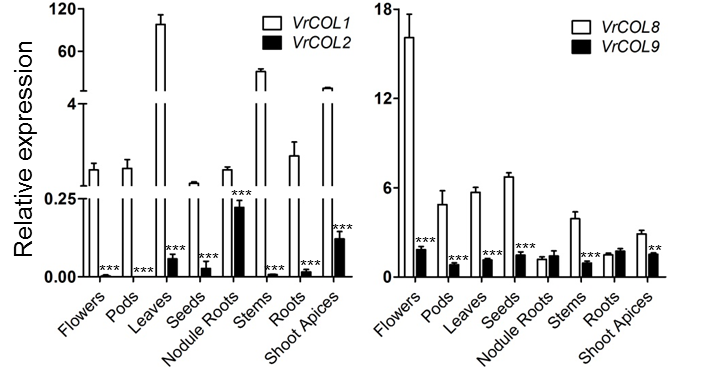


**Supplementary Figure S3 Relative expression levels of *VrCOL* duplicated genes in different tissues.** The expression level of *VrCOL1* in flowers was set as 1, and the others were adjusted accordingly. *** and ** are significantly different at P < 0.001 and P < 0.01, respectively, compared with the expression levels in its duplicated gene.


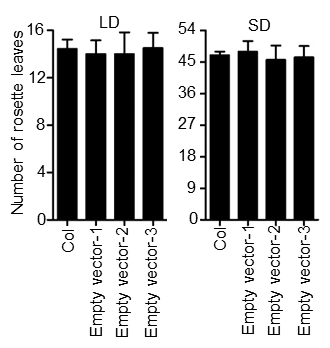


**Supplementary Figure S4 The rosette leaf numbers of empty vector transgenic lines and wild-type plants grown under LD and SD conditions.**


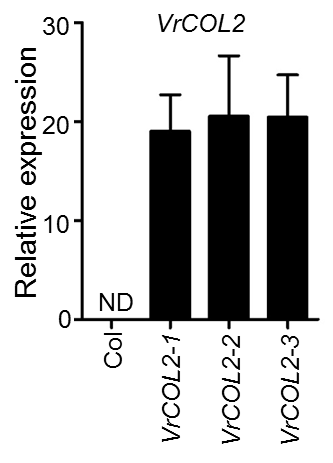


**Supplementary Figure S5 Expression analysis of *VrCOL2* in *VrCOL2* transgenic lines and wild-type *Arabidopsis* measured by qRT-PCR.** The leaves of transgenic and wild-type plants grown under LD conditions were sampled 5 h after lights-on for qRT-PCR analysis. Gene expression levels were normalized to an Actin gene from Arabidopsis. ND, not detected.
